# Comparison of Protective Effects of Phenolic Acids on Protein Glycation of BSA Supported by In Vitro and Docking Studies

**DOI:** 10.1155/2023/9984618

**Published:** 2023-07-18

**Authors:** Marzieh Rashedinia, Zeinab Rasti Arbabi, Razieh Sabet, Leila Emami, Alireza Poustforoosh, Zahra Sabahi

**Affiliations:** ^1^Food and Supplements Safety Research Center, Shiraz University of Medical Sciences, Shiraz, Iran; ^2^Medicinal Plants Processing Research Center, Shiraz University of Medical Sciences, Shiraz, Iran; ^3^Department of Pharmacology and Toxicology, School of Pharmacy, Shiraz University of Medical Sciences, Shiraz, Iran; ^4^Department of Medicinal Chemistry, Faculty of Pharmacy, Shiraz University of Medical Sciences, Shiraz, Iran; ^5^Pharmaceutical Sciences Research Center, Shiraz University of Medical Sciences, Shiraz, Iran; ^6^Medicinal and Natural Products Chemistry Research Center, Shiraz University of Medical Sciences, Shiraz, Iran

## Abstract

Several diabetic complications are associated with forming advanced glycation end products (AGEs). Different chemical and natural compounds are able to prevent the development of these products. In this study, glycosylation was induced as a model by incubating bovine serum albumin (BSA) with glucose. Consequently, BSA was treated with glucose and different concentrations (1.25, 2.5, and 5 *μ*M) of syringic acid, gallic acid, ellagic acid, ferulic acid, paracoumaric acid, and caffeic acid for 4 and 6 weeks. Biochemical experiments comprise measurements of fluorescent AGEs, protein carbonyl contents, total thiol, hemolysis tests, and also malondialdehyde (MDA) levels in RBC. These demonstrated the antiglycating mechanism of these phenolic acids. Most of the phenolic acids used in this study reduced MDA levels and protected thiol residues in protein structures. They also inhibited the formation of fluorescent AGEs and RBC lysis, except gallic acid. Moreover, ferulic acid, paracoumaric acid, and caffeic acid proteins significantly prevent carbonylation. Molecular docking and simulation studies showed that ellagic, caffeic, gallic, and syringic acids could interact with lysine and arginine residues in the active site of BSA and stabilize its structure to inhibit the formation of AGEs. Our results suggest that phenolic acid could be used as a potential phytochemical against protein glycation and related diabetic complications.

## 1. Introduction

The nonenzymatic glycation of proteins refers to different spontaneous reactions between reducing sugars and amine groups. In the glycation process, elevated levels of glucose induce covalent adducts with plasma proteins. The products of these glycation reactions are advanced glycation end products (AGEs). In these processes, protein structures change and they lose their biological activities [1].

Lipid and amino acid oxidation may be nonglucose sources of AGEs production [2].

AGEs can stimulate intracellular oxidative stress by producing reactive oxygen species (ROS). Conversely, the increased ROS level is able to promote the production of AGEs, so there is a strong relationship between oxidative stress and AGEs [3]. The accumulation of AGEs and oxidative stress accelerates ageing process and leads to different diabetic complications such as retinopathy, neuropathy, nephropathy, protein denaturation, and inflammation [1, 4, 5]. Also, cardiovascular disease, Alzheimer's disease, cystic fibrosis, non-B or non-C hepatocellular carcinoma, schizophrenia, blindness, cataracts, and some types of cancer are associated with increased AGEs levels [2, 6].

Inhibiting the formation of AGEs by different antiglycating compounds is considered an effective strategy for reducing AGE-mediated diabetic complications [1, 7]. Synthetic compounds including aminoguanidine (AG), pyridoxamine, N-phenacylthiazolium bromide, and amlodipine have been known as effective inhibitors of protein glycation. Nevertheless, their different side effects limited their clinical applications [1].

Some drugs such as metformin, pioglitazone, angiotensin receptor blockers, inhibitors of angiotensin-converting enzyme, and pentoxyfylline were also determined to inhibit AGE formation. Moreover, vitamin C and E, metal ion chelators (desferoxamine and penicillamine), and aspirin can inhibit protein glycation [2].

Beside chemical compounds, natural products have also been considered as rich sources of natural compounds to prevent AGEs-related complications [2]. Different glycation assays exhibited that polyphenolic compounds possess inhibitory effects on the glycation reaction [2, 8–10].

Phenolic acids, both hydroxybenzoic acid (HBA) and hydroxycinnamic acid (HCA) derivatives are known phenolic compounds. Previous *in vitro* experiment confirmed that HBA and HCA groups of phenolic acids were able to prevent of the early stage of glycation [11, 12].

Phenolic acids are also potent antioxidants [13] and have antidiabetic effects by increasing the release of insulin from pancreas and/or regenerated *β*-cells, restoring insulin sensitivity and raising the consumption of glucose by peripheral tissues [14, 15]. According to the study by Gugliucci et al., the antiglycation effect of yerba maté (*Ilex paraguariensis*) water extracts was related to present of chlorogenic acid and caffeic acid as the main compounds in maté tea [16]. Also, chlorogenic [17], caffeic [7], ferulic [18], gallic, and cinnamic acids [19] were introduced in previous reports as antioxidants [20] and proper candidates in the prevention of AGEs.

The present study focuses on the evaluation of the antiglycation capacity of syringic (Syr), gallic (Gal), ellagic (El), ferulic (Fer), para-coumaric (Com), and caffeic (Caf) acids.

We compared the protective effects of these natural compounds against AGEs production by different methods such as bovine serum albumin (BSA) glycation, measurement of fluorescent AGEs, determination of protein carbonyl contents, assay of total thiol, hemolysis of red blood cell (RBC) test, and determination of MDA level in RBC. Moreover, molecular docking was carried out to define the binding mode, interaction type, and free binding energy of these compounds in the active site of the BSA protein.

## 2. Materials and Methods

### 2.1. Chemicals

All the chemicals used in this experiment were purchased from Sigma-Aldrich (St. Louis, USA) and Merck Company (Darmstadt, Germany), the highest grade commercially available.

### 2.2. Bovine Serum Albumin (BSA) Glycation

The glycation reaction was performed based on a previous method with slight modifications [21]. BSA (10 mg/mL) was dissolved in PBS (20 mM, pH 7.4) and incubated with glucose (1.1 M) in phosphate buffer and sodium azide 0.2%). The phenolic acids, gallic acid (Gal), syringic acid (SYR), ellagic acid (Ela), ferulic acid (Fer), p-coumaric acid (Com), and caffeic acid (Caf) stock solution was prepared and dissolved in the least amount of DMSO; then, different concentrations of phenolic acids were added to the BSA mixture with final concentrations (1.25, 2.5, and 5 *μ*M) and were incubated at 37°C for 4 and 6 weeks. A control reaction was without phenolic acid and represented 100% glycation. The effect of the final concentration of DMSO as a solvent for phenolic acids was evaluated. At the end of the incubation times, the reaction mixtures were frozen at −20°C before the next analysis.

### 2.3. Measurement of Fluorescent AGEs

The glycation process leads to the formation of fluorescent AGEs. So, these products could be measured at an excitation wavelength of 360 nm and an emission maximum of 460 nm. Fluorescence was measured using a FLUOstar Omega microplate reader (BMG Labtech, Germany). The results were expressed as the fluorescent intensity [18].

### 2.4. Determination of Protein Carbonyl Contents

According to the previous method [18], the reaction mixtures were incubated with 10 mM DNPH (at room temperature for 10 min in the dark). Then, TCA was added to precipitate proteins in the mixtures and centrifuged at 6000*g* at 4°C for 10 min. The protein pellets were washed with acetone and centrifuged (6000*g* at 4°C for 15 min). The supernatants were discarded after each centrifugation. The final protein pellets were dissolved in a protein solvent. The absorbance was measured at 375 nm. The results were expressed as nmol/mg protein.

### 2.5. Assay of Total Thiol

Thiol concentrations were measured by using 5, 5′-dithionitrobenzoic acid (DTNB) [22]. Briefly, 200 *μ*L of freshly prepared DTNB in phosphate buffer was added to 100 *μ*L of diluted sample or buffer. Reduced glutathione was used to prepare standard curves. Release of the 5-thiobenzoate anion (yellow complex) was measured at 412 nm using a ELISA plate reader [23].

### 2.6. Hemolysis Test

Blood samples from rats (3 ml) were collected in tubes containing EDTA, and then, 5 ml phosphate buffer was added. 100 *μ*l of diluted blood mixed with 300 *μ*l of phenolic acids was incubated for 24 h at 37°C. The release of hemoglobin was determined after centrifugation (6000*g* for 15 min) by photometric analysis of the supernatant at 540 nm. The experiments were performed in triplicate and H_2_O_2_ used as a positive control [24].

### 2.7. Determination of the Malondialdehyde (MDA) Level in RBC

The RBC and phenolic acid samples were incubated at room temperature for 24 h and centrifuged. The supernatants reacted with thiobarbituric acid in a boiling water bath for 45 min. After cooling, the absorbance was read at 532 nm [25].

### 2.8. Docking Study

AutoDock Tools package (1.5.6) (ADT) (https://mgltools.scripps.edu/) software was used to perform the molecular docking studies. The 3-D X-ray crystal structure of BSA (PDB ID: 4F5S) at 2.47 Å resolution was selected from the RCSB Protein Databank (https://www.rcsb.org). Before docking, the cognate ligand and water molecules were removed, missing hydrogen was added to atoms, and finally saved in PDBQT format. The HyperChem software was used to generate and minimize (by Molecular Mechanics MM+ and then semiempirical AM1 methods) 3D structures of ligands. PDBQT formats of the ligands were achieved by adding Gasteiger charges and adding the degree of torsions. The docking procedure was performed in a grid box with a size of 75 × 75 × 75 Å with 0.375 grid spacing and a center of *x* = 3.41, *y* = 27.983, *z* = 106.347 using AutoDock Vina (1.1.2) using an in-house batch script (DOCKFACE) [26, 27]. The images showing the interactions of the binding complexes were created using discovery studio 2017 R2 client (https://accelrys.com).

### 2.9. Molecular Dynamics Simulation (MD)

The interactions between ellagic acid and BSA (PDB ID: 4F5S) were further evaluated in a dynamic situation. Desmond Schrödinger was used for MD simulation [28]. The complex system obtained from the docking calculation was used for the MD [29]. The simulation was performed in an orthorhombic box, and the solvent model of transferable intermolecular potential with 3 points (TIP3P) was chosen for the simulation [30]. The proper number of Na+/Cl− ions with a salt concentration of 0.15 M was used to neutralize the system employing the system setup of Schrödinger [31]. The simulation was then accomplished for 100 ns with the default relaxation protocol of the software and the constant number of atoms, pressure, and temperature (NPT) ensemble [32]. The Nose-Hoover protocol was used to set the temperature to 310.15 K (37°C), and the pressure was adjusted to 1 atm employing isotropic scaling [33].

### 2.10. Statistical Analysis

The data are presented as mean ± SEM. All the calculations were carried out by SPSS 16.0 software. A One-way ANOVA with Dunnett's test was used for analysis. The values with *p* < 0.05 were considered statistically significant. The GraphPad Prism 5.0 software was used for plotting graphs.

## 3. Results

### 3.1. The Effect of Phenolic Acids on BSA-Derived Fluorescence

**Figure 1:**
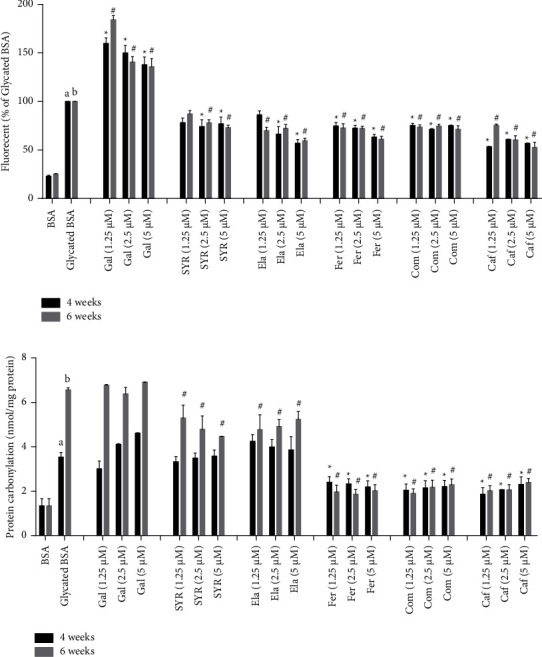
(a) The effect of phenolic acids on inhibition of fluorescence AGEs. The fluorescent intensity was monitored at excitation and emission wavelengths of 360 and 460 nm. (b) The effect of phenolic acids on protein carbonyl contents. The results are expressed as mean ± SEM (*n* = 3). *a*, *b* significant difference *p* < 0.05 compared with BSA after 4 and 6 weeks incubation times.^*∗*^*p* < 0.05 compared with glycated BSA after 4 weeks incubation time, ^#^*p* < 0.05 compared with glycated BSA after 6 weeks incubation time. BSA: bovine serum albumin, Gal: gallic acid, SYR: syringic acid, Ela: ellagic acid, Fer: ferulic acid, Com: p-coumaric acid, Caf: caffeic acid.

Since AGEs have fluorescent properties, the method of fluorescence spectroscopy was used to evaluate the extent of AGEs formation. The fluorescence intensity of BSA and glucose in the presence or absence of phenolic acids is presented in Figure 1(a). In both incubation times, the fluorescence intensity of the mixture of glucose and BSA increased significantly compared with BSA. Whereas, the addition of Fer, Com, and Caf (1.25, 2.5, and 5 *μ*M) decreased the fluorescence intensity in comparison with glycated BSA (*p* < 0.05), which showed the ability of those phenolic acids to inhibit AGEs formation. The 2.5 and 5 *μ*M concentrations of SYR and Ela exhibit the same trend, while Gal do not have inhibitory effect against glycation-induced fluorescence intensity. Conversely, Gal treatment leads to an increase fluorescence intensity compared to glycated BSA (*p* < 0.05) after 4- and 6-weeks incubation time. Also, the final concentration of DMSO as a solvent for phenolic acids had no effect on experiments (data not shown).

### 3.2. The Effect of Phenolic Acids on Protein Carbonyl Contents

**Figure 2:**
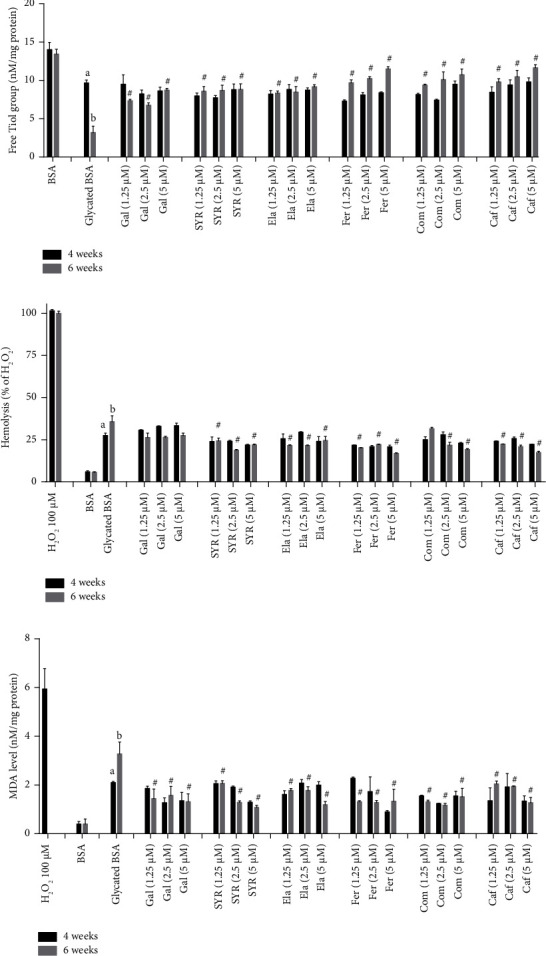
(a) The protection effect of phenolic acids on thiol groups in protein glycation. (b) The protection effect of phenolic acids on RBC hemolysis. (c) The protection effect of phenolic acids on lipid peroxidation in RBC. The results are expressed as mean ± SEM (*n* = 3). *a*, *b* significant difference *p* < 0.05 compared with BSA after 4 and 6 weeks incubation times. ^#^*p* < 0.05 compared with BSA after 6 weeks incubation time. H_2_O_2_ used as positive control. BSA: bovine serum albumin, Gal: gallic acid, SYR: syringic acid, Ela: ellagic acid, Fer: ferulic acid, Com: p-coumaric acid, Caf: caffeic acid.

The effect of phenolic acids on protein carbonyl contents is presented in Figure 2(b). The levels of protein carbonyl in glycated BSA significantly increased after 4 and 6 weeks of incubation. While, addition of Fer, Com, and Caf (1.25, 2.5 and 5 *μ*M) blocked protein carbonyl content levels of BSA significantly (*p* < 0.05) after 4 and 6 weeks of incubation. Gal, SYR and Ela were unable to inhibit of protein carbonilation after 4 weeks incubation. However, SYR and Ela decreased protein carbonylation in glycated BSA significantly after 6 weeks of incubation (*p* < 0.05).

### 3.3. The Effect of Phenolic Acids on Protein Thiol

The thiol groups of cysteine residues are one of the chief targets of protein glycation. Glucose-induced glycated BSA showed a significant decrease in free-SH groups in comparison with BSA significantly (*p* < 0.05) (Figure 2(a)). The free-SH content of glycated BSA in the presence of 1.25, 2.5, and 5 *μ*M concentrations of Gal, Caf, Com, Ela, Fer, and SYR protects against the significantly reduction of thiol groups of BSA, only after 6 weeks incubation time (*P* < 0.05).

### 3.4. The Effect of Phenolic Acids on RBC Hemolysis

H_2_O_2_ induces oxidative damage in the cell membrane and leads to RBC hemolysis. Our result showed that glycated BSA showed significantly increased RBC hemolysis in comparison with BSA (*p* < 0.05). The RBC hemolysis was inhibited in the presence of 1.25, 2.5, and 5 *μ*M concentrations of Caf, Com, Ela, Fer, and SYR phenolic acids(6 weeks) (*p* < 0.05). Gal did not show any protective effects at both incubation times (Figure 2(b)).

### 3.5. The Effect of Phenolic Acids on MDA Level in RBC

The effects of phenolic acids on lipid peroxidation in RBC are shown in (Figure 2(c)). The level of MDA increased in glycated BSA significantly (*p* < 0.05). Treatment of groups with 1.25, 2.5, and 5 *μ*M of phenolic acids (Gal, Caf, Com, Ela, Fer, and SYR) decreased the amount of MDA in 6 weeks incubation time significantly (*p* < 0.05).

### 3.6. Docking Study

**Figure 3:**
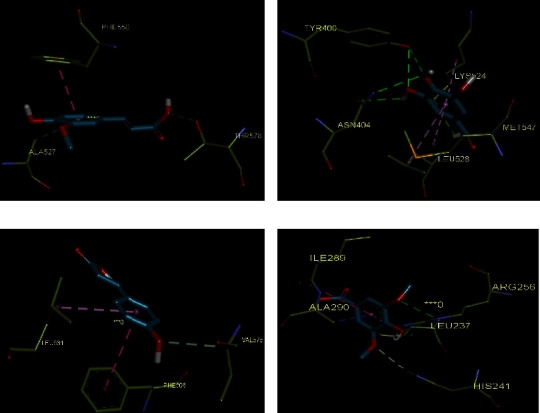
The interactions of compounds Fer (a), Gal (b), Com (c), and SYR (d) with BSA (PDB: 4FS5). The compounds are showed as blue sticks. Hydrogen bonding and hydrophobic and electrostatic interactions are showed as green, pink, and orange, respectively.

BSA is a serum albumin protein contains 583 amino acid residues. Molecular modeling was performed to understand the binding mode, interaction type, and free binding energy of the studied compounds in the active site of the BSA protein; the key interaction model for the studied compounds is displayed in Table 1. The range of free binding energy values was observed between −6.3 (Gal) and −8.7 (Ela), the binding mode of Fer, Gal, Com, and SYR in the active site of the BSA is provided in Figures 3(a)–3(d).

As depicted in Figure 3, the phenyl ring of the Fer binds to the Phe 527 and Ala 550 via pi–pi and pi-alkyl interactions. Also, hydroxyls group of Fer interacts through a hydrogen bond with Thr 578. Gal interacted with BSA with hydrogen binding to the carbonyl oxygen of Tyr 400, Asn 404, and Lys 524 and some hydrophobic interactions with the residues Leu 527, Met 547, and Lys 524 were observed. The binding mode of Com was summarized with carbon hydrogen bond interaction with Val 575 and hydrophobic interaction with hydrophobic amino acid resides such as Leu 531 and Phe 506. In SYR, there are hydrogen bond interactions with Arg 256, His 241, and Ala 290, as well as some hydrophobic interactions with Leu237 and Ile 289.

**Figure 4:**
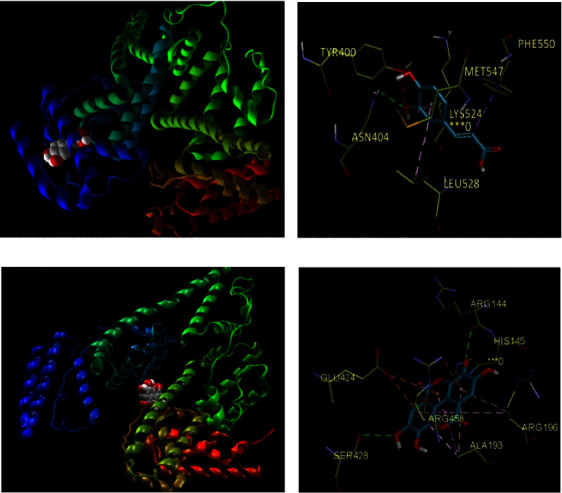
3D presentations of the molecular docking results. (a) The minimum energy conformation of caffeic acid-BSA complex obtained from molecular docking. (b) Interactions of caffeic acid with the residues in the binding site of the BSA receptor (4FS5). 3D presentations of the molecular docking results. (c) The minimum energy conformation of ellagic acid-BSA complex obtained from molecular docking. (d) Interactions of ellagic acid with the residues in the binding site of the BSA receptor (4FS5).

The phenyl ring in Caf formed a pi-alkyl with Met 547, Lys 524, and Leu 528, the double bond of the acrylic acid moiety of Caf formed a pi-sigma interaction with Phe 550; and a hydrogen bond between the OH group and Tyr 400 and Asn 404 was also observed (Figures 4(a)) and 4(b).

Figures 4(c) and 4(d) indicate that Ela had close contact interactions with several of the amino acid residues; the chromene moiety of Gal interacted with Arg 458 and Glu 424 through pi-cation and pi-anion interactions, respectively. Also, it displayed that Gal combined with Arg 144 and Ser 428 through hydrogen bond interaction and pi-alkyl and pi-sigma interaction with Arg 196, Ala 193, and Arg 458. These interactions with several arginines might play a role in its enhanced binding energy compared to the other studied compounds.

### 3.7. MD Simulation

**Figure 5:**
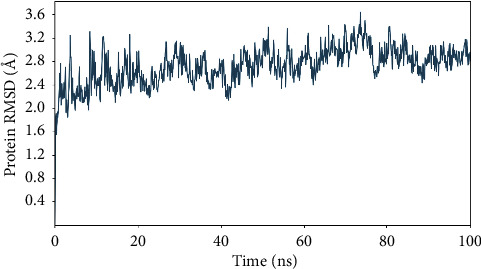
The RMSD values of MD simulation for protein, which are converged at about 2.8 Å.

**Figure 6:**
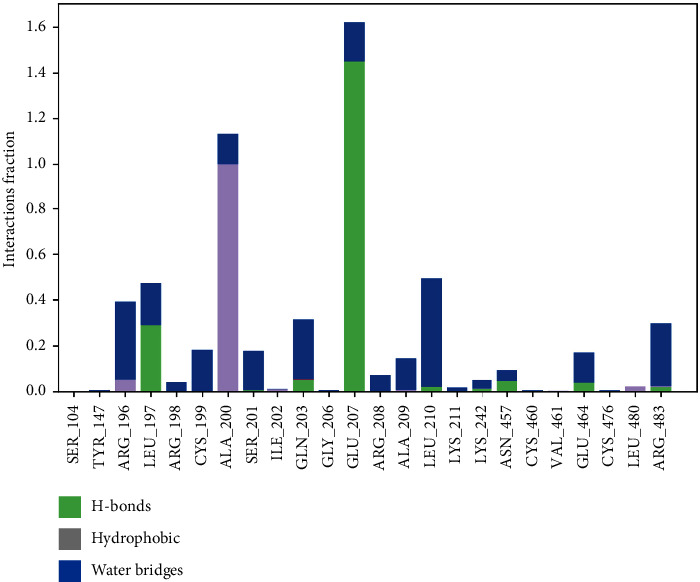
The interactions constructed between the protein and ellagic acid during the MD simulation.

**Figure 7:**
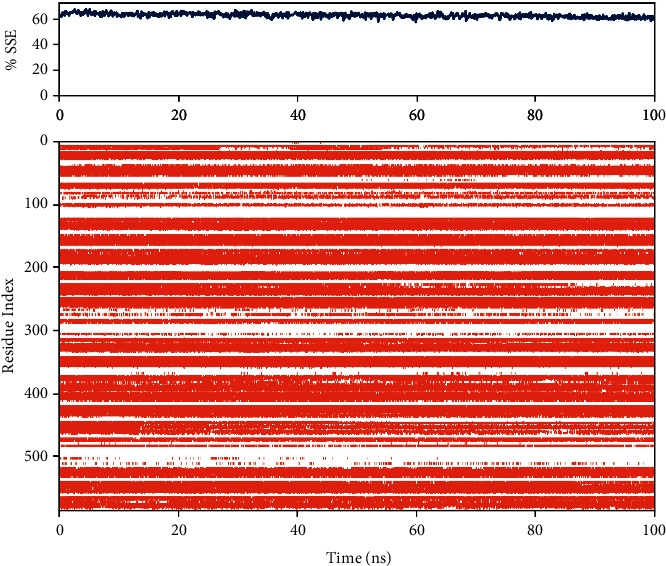
The percent variation of SSE for the protein during the MD simulation. The red and blue regions indicate the alpha helices and beta strands, respectively.

Considering that the highest free binding energy in the active site of BSA protein was related to Ela, the ligand-protein complex of ellagic acid was further evaluated using the MD simulation for 100 ns. The RMSD value of the protein in the simulation is presented in Figure 5. As could be seen, the fluctuations for the protein RMSD converged at about 2.8 Å that indicates the stability of the system after simulation time. The ligand-protein interactions between tyrosinase and ellagic acid during 100 ns are presented in Figure 6. The residues with the highest interaction fraction are Glu207, Ala200, Leu197, Leu210, Arg196, and Arg483. The major interactions constructed by Glu207 and Ala200 during the simulation are hydrogen bond and hydrophobic contacts, respectively. The major interaction is the Arg196 water bridge. Arg196 is a key residue of BSA and targeting this residue can inhibit the formation of AGEs, as reported by Awasthi et al. [34]. Arg483 is another residue with water bridges interaction during the simulation time. Like Arg196, this residue is important in BSA for inhibiting AGEs formation [35]. The details of other interactions between ellagic acid and tyrosinase during the simulation time are presented in Figure 6. The percent of secondary structure elements (SSE) during the MD simulation is presented in Figure 7. As could be seen in Figure 7, the alpha-helical structures (red zone) of the protein have not experienced considerable changes during the simulation. This outcome indicates the stability of the protein's secondary structure and the validity of the simulation.

## 4. Discussion

AGEs formation is the result of the Maillard reaction or nonenzymatic glycation reaction. This process comprises different steps, so it takes several days or several weeks [11]. In the initial phase of this reaction reducing sugars, such as glucose, bind to the terminal amino groups of proteins, nucleic acids, or phospholipids and produce unstable Schiff bases. These bases become more stable and are known as keto-amines (Amadori products). Schiff bases are susceptible to oxidation, form free radicals, and produce active carbonyl derivatives. In the advanced phase, dicarbonyl compounds react with arginine and lysine residues to produce AGEs [11].

In this study, the reaction of glucose with BSA used as a model for investigating the protective effects of phenolic acid on protein glycation. Treatment of BSA with Fer, Com, Caf, SYR, and Ela decreased the fluorescence intensity, which showed the ability of those phenolic acids to inhibit AGEs formation. Moreover, Fer, Com, and Caf can decrease protein carbonyl content levels of BSA significantly. While SYR and Ela were unable to inhibit protein oxidation after 4 weeks of incubation, they decreased protein carbonylation significantly after 6 weeks. Whereas, Gal did not show inhibitory effects on glycation-induced fluorescent intensity and was also unable to protect the protein carbonylation process.

The thiol groups of cysteine are one of the chief targets of protein glycation. Therefore, glycation leads to the formation of disulfide bonds. All phenolic acids used in this study were able to inhibit AGEs formation by reducing the oxidation of free thiol groups in BSA after 6 weeks.

Various mechanisms can be applied to decrease AGEs production. During the early stage (Maillard reaction) a large amount of free radicals are synthesized. Also, Schiff bases are susceptible to producing free radicals and reactive carbonyl groups. So, one strategy is scavenging free radicals to reduce oxidative stress and decrease the production of reactive carbonyl group [11]. Previous studies revealed that scavenging ability of phenolic acids was related to the inhibition of AGEs formation [35–37]. The other mechanism of phenolic compounds to prevent oxidation is ion chelating activities and blocking the self-oxidation of sugars, Amadori products and reactive carbonyl species. In addition, natural phenolic compounds trap the reactive carbonyl intermediates such as methylglyoxal (MGO) [38].

Phenolic acids are aromatic acid derivatives, so a phenolic ring and an organic carboxylic acid function (C6-C1 skeleton) are denoted. Two main types of phenolic acids, hydroxybenzoic acids and hydroxycinnamic acids, are derived from benzoic and cinnamic acids, respectively [37, 39]. Previous study showed that the number of hydroxyl groups in their structure is a determinative factor in phenolic acid-HSA (human serum albumin) binding affinity. It seems that the presence of more hydroxyl groups in their structures may diminish affinity for HSA. Since the addition of hydroxyl groups reduces the hydrophobic property of phenolic acids and consequently decreases their ability to enter hydrophobic sections of HAS [37, 40].

This is correlated with our data which showed that Gal was unable to prevent protein carbonylation and AGEs production. Since Gal has three hydroxyl groups in its structure, it exhibits free radical scavenging activity, but these groups may reduce its affinity with BSA. While Fer, Com, Caf, and SYR have less number of hydroxyl groups.

Beside the number of hydroxyl groups, the position of this group on the benzene ring also plays an important role in their affinities with HAS. The hydroxyl group at the 2-position and the 4-position of the benzene ring had a positive and negative effect on the affinities for HSA, respectively [37].

Zhang et al. suggested that the presence of two methoxy substituents on the aromatic ring leads to steric hindrance, so the affinity of the protein binding site of sinapic acid is lower than that of caffeic acid and p-coumaric acid [37, 41]. Since SYR has two methoxy groups on the aromatic ring, the protective effect of this phenolic acid against protein carbonylation was less than that of Fer, Com, and Caf, and it was also revealed after 6 weeks of incubation time, not after 4 weeks.

Other studies showed that arginine and lysine amino acids are considered sites of protein glycation [7]. Our docking study showed that Ela, Caf, Ga, and SYR interacted with the two amino acid residues of BSA to stabilize its structure, so the formation of AGEs could be inhibited.

Among these phenolic acids, Ela showed more interaction numbers and diversity and the most binding interactions between Caf and Com and BSA were hydrophobic.

In a previous report, it was shown that Caf had more antiglycation effects in comparison to chlorogenic acid. It seems that hydrophobicity or their overall size and polarity were determinative factors in their behaviors [42].

Erythrocytes are very vulnerable to the oxidative damage due to the high cellular concentration of hemoglobin and oxygen. According to our results, RBC hemolysis was inhibited by glycated BSA treated with Caf, Com, Ela, Fer, and SYR phenolic acids. Hyperglycemia leads to oxidative stress by increasing the ROS level [14], which leads to the oxidative degradation of biomolecules, particularly the lipid membrane. Thus, lipid peroxidation is a cellular marker of membrane oxidative damages [43, 44].In this study, phenolic acid comprise Caf, Com, Ela, Fer, and SYR-reduced MDA level of RBC in oxidative stress condition. It seems that their hemolysis preventive ability was related to lipid peroxidation inhibition activity. Moreover, these compounds were able to preserve thiol residues. Consequently, the protection of SH groups in membrane proteins can preserve membrane stability [45].

In consistence with these observations, a previous study reported that Fer inhibited lipid peroxidation in RBC exposed to glucose. Also, Fer increased Na^+^/K^+^-ATPase activity in the RBC membrane which was reduced significantly in hyperglycemia [43].

## 5. Conclusion

Based on our results, phenolic acids (syringic, gallic, ellagic, ferulic, paracoumaric, and caffeic acids) reduced MDA levels and protected thiol residues in the protein structure. They also inhibited the formation of AGEs.

Among them, ferulic acid, paracoumaric acid, and caffeic acid protein prevent carbonylation significantly. Also, molecular docking and simulation studies showed that ellagic, caffeic, gallic, and syringic acids could interact with lysine and arginine residues in the active site of BSA and stabilize its structure to inhibit the formation of AGEs.

Therefore, future studies would explain the effects of phenolic acid on the production of AGEs in vivo and consider the application of polyphenols in glycation-related diseases.

## Figures and Tables

**Table 1:** Molecular docking results for the interaction between phenolic acids and BSA.

Compound	Amino acid residue	Interaction type	Distance (Å)	Binding energy (kcal mol^−1^)
Caffeic acid	Tyr 400	Hydrogen bond	2.91	−7.2
	Asn 404	Hydrogen bond	2.59	
	Leu 508	Hydrophobic	494	
	Lys 542	Hydrophobic	4.50	
	Met 547	Hydrophobic	4.40	
	Phe 550	Hydrophobic	3.85	

Ellagic acid	Glu 424	Electrostatic	4.71, 4.55	−8.7
	Ser 428	Hydrogen bond	3.23	
	Ala 193	Hydrophobic	3.81, 4.34, 5.29, 5.39	
	Arg 196	Hydrophobic	5.39, 4.79	
	Arg 458	Electrostatic, hydrophobic	4.9, 4.15, 3.51, 4.27	
	His 145	Hydrophobic	2.0	
	Arg 144	Hydrogen bond	2.94	

Ferulic acid	Ala 527	Hydrogen bond	2.90	−6.9
	Phe 550	Hydrophobic	3.92	
	Thr 578	Hydrophobic	4.20	

Gallic acid	Tyr 400	Hydrogen bond	3.05, 3.22	−6.3
	Asn 404	Hydrogen bond	2.98, 3.23	
	Leu 527	Hydrophobic	2.19, 4.39	
	Met 547	Hydrophobic	4.32	
	Lys 524	Hydrogen bond, hydrophobic	2.36, 4.58	

p-Coumaric acid	Leu 531	Hydrophobic	5.01	−7.1
	Phe 506	Hydrophobic	4.52	
	Val 575	Hydrogen bond	3.78	

Synergic acid	Ile 289	Hydrophobic	4.78	−6.5
	Ala 290	Hydrogen bond	3.58	
	His 241	Hydrogen bond	3.58	
	Leu 237	Hydrophobic	4.99	
	Arg 256	Hydrogen bond	3.03, 3.13	

## Data Availability

The data used to support the findings of this study are included within the article.
